# Direct-to-consumer DNA testing of 6,000 dogs reveals 98.6-kb duplication associated with blue eyes and heterochromia in Siberian Huskies

**DOI:** 10.1371/journal.pgen.1007648

**Published:** 2018-10-04

**Authors:** Petra E. Deane-Coe, Erin T. Chu, Andrea Slavney, Adam R. Boyko, Aaron J. Sams

**Affiliations:** 1 Embark Veterinary, Inc., Boston, Massachusetts, United States of America; 2 Department of Biomedical Sciences, College of Veterinary Medicine, Cornell University, Ithaca, New York, United States of America; Stanford University School of Medicine, UNITED STATES

## Abstract

Consumer genomics enables genetic discovery on an unprecedented scale by linking very large databases of personal genomic data with phenotype information voluntarily submitted via web-based surveys. These databases are having a transformative effect on human genomics research, yielding insights on increasingly complex traits, behaviors, and disease by including many thousands of individuals in genome-wide association studies (GWAS). The promise of consumer genomic data is not limited to human research, however. Genomic tools for dogs are readily available, with hundreds of causal Mendelian variants already characterized, because selection and breeding have led to dramatic phenotypic diversity underlain by a simple genetic structure. Here, we report the results of the first consumer genomics study ever conducted in a non-human model: a GWAS of blue eyes based on more than 3,000 customer dogs with validation panels including nearly 3,000 more, the largest canine GWAS to date. We discovered a novel association with blue eyes on chromosome 18 (*P* = 1.3x10^-68^) and used both sequence coverage and microarray probe intensity data to identify the putative causal variant: a 98.6-kb duplication directly upstream of the Homeobox gene *ALX4*, which plays an important role in mammalian eye development. This duplication is largely restricted to Siberian Huskies, is strongly associated with the blue-eyed phenotype (chi-square *P* = 5.2x10^-290^), and is highly, but not completely, penetrant. These results underscore the power of consumer-data-driven discovery in non-human species, especially dogs, where there is intense owner interest in the personal genomic information of their pets, a high level of engagement with web-based surveys, and an underlying genetic architecture ideal for mapping studies.

## Author summary

The genetic underpinnings of many phenotypic traits in domestic dogs remain undiscovered. Although two genetic loci are known to underlie blue eye color in dogs, these do not explain all cases of blue eyes. By examining > 3,000 dogs from the Embark Veterinary, Inc. customer database, representing the first genome-wide association study (GWAS) driven by consumer genomics in dogs and the largest dog GWAS cohort to-date, we have shown that a region of canine chromosome 18 carrying a tandem duplication near the *ALX4* gene is strongly associated with blue eye color variation, primarily in Siberian Huskies. We also provide evidence that this duplication is associated with blue eye color in non-merle Australian Shepherds. While beyond the scope of this work, future studies of the functional mechanism underlying this association may lead to discovery of a novel pathway by which blue-eyes develop in mammals. These results highlight the power and promise of consumer-data-driven discovery in non-human species.

## Introduction

Humans have been exerting multifarious selection on dogs since their domestication from wolves, including strong natural selection during adaptation to a domesticated lifestyle followed by intense artificial selection during modern breed formation [1–3]. One unintended consequence of this selection is that the canine genome now encodes dramatic phenotypic diversity highly amenable for genetic mapping, with moderate genome-wide divergence between breeds except near loci under selection [4–6] and long tracts of linkage disequilibrium that can be effectively scanned with microarrays [7]. Genetic discoveries in dogs benefit breeding efforts and animal welfare, and they are valuable for translational studies in humans because dogs and humans exhibit many analogous physical traits, behaviors, and diseases in a shared environment [5, 8].

In humans, blue eyes first arose in Europeans [9] and may have been favored by sexual selection due to an aesthetic preference for rare phenotypic variants [10], as an informative recessive marker of paternity [11], and/or as a by-product of selection for skin de-pigmentation to increase UVB absorption [12]. Whatever the cause, this selection has acted on the regulatory machinery of *OCA2* (Oculocutaneous Albinism II Melanosomal Transmembrane Protein), which controls transport of the melanin precursor tyrosine within the iris [13, 14]. Because blue eyes result from reduced melanin synthesis, other mutations affecting melanocyte and melanosome function in the retinal pigment epithelium (RPE) can also recapitulate the phenotype [15].

In dogs, blue eyes are iconic of the Siberian Husky, a breed of northern latitudes. Prized among breeders, it is not known whether blue eyes confer adaptive benefits for high latitude dogs as has been hypothesized for humans, and the genetic basis has not yet been discovered. According to breeders, blue eyes in Siberian Huskies are a common and dominant trait, including solid blue and complete heterochromatism (one blue and one brown eye), whereas blue eyes appear to be a rare and recessive trait in breeds like the Border Collie, Pembroke Welsh Corgi, and Old English Sheepdog. The only genetic factors known to produce blue eyes are two cases associated with coat coloration: “Merle” and “piebald” dogs have patchy coat colors due to mutations in Premelanosome Protein (*PMEL17*) and Melanogenesis Associated Transcription Factor (*MITF*) that can lead to one or two blue eyes, or slices of sectoral heterochromia, when de-pigmented regions extend across the face [16, 17]. *PMEL* is regulated by *MITF*, the master regulator of melanocyte development [18]. Rarely, non-merle Australian Shepherds have unexplained cases of solid blue eyes or complete heterochromia, as in huskies, and the genetic basis of this trait is similarly unknown [19].

We employed a novel genomic resource—a panel of 6,070 dogs genetically tested on a high-density 214,661-marker platform, with owners that had contributed phenotype data via web-based surveys and photo uploads—to examine the genetics of blue eyes in a diverse panel of purebred and mixed-breed dogs.

## Results

### A novel association with blue eye color near *ALX4*

**Fig 1:**
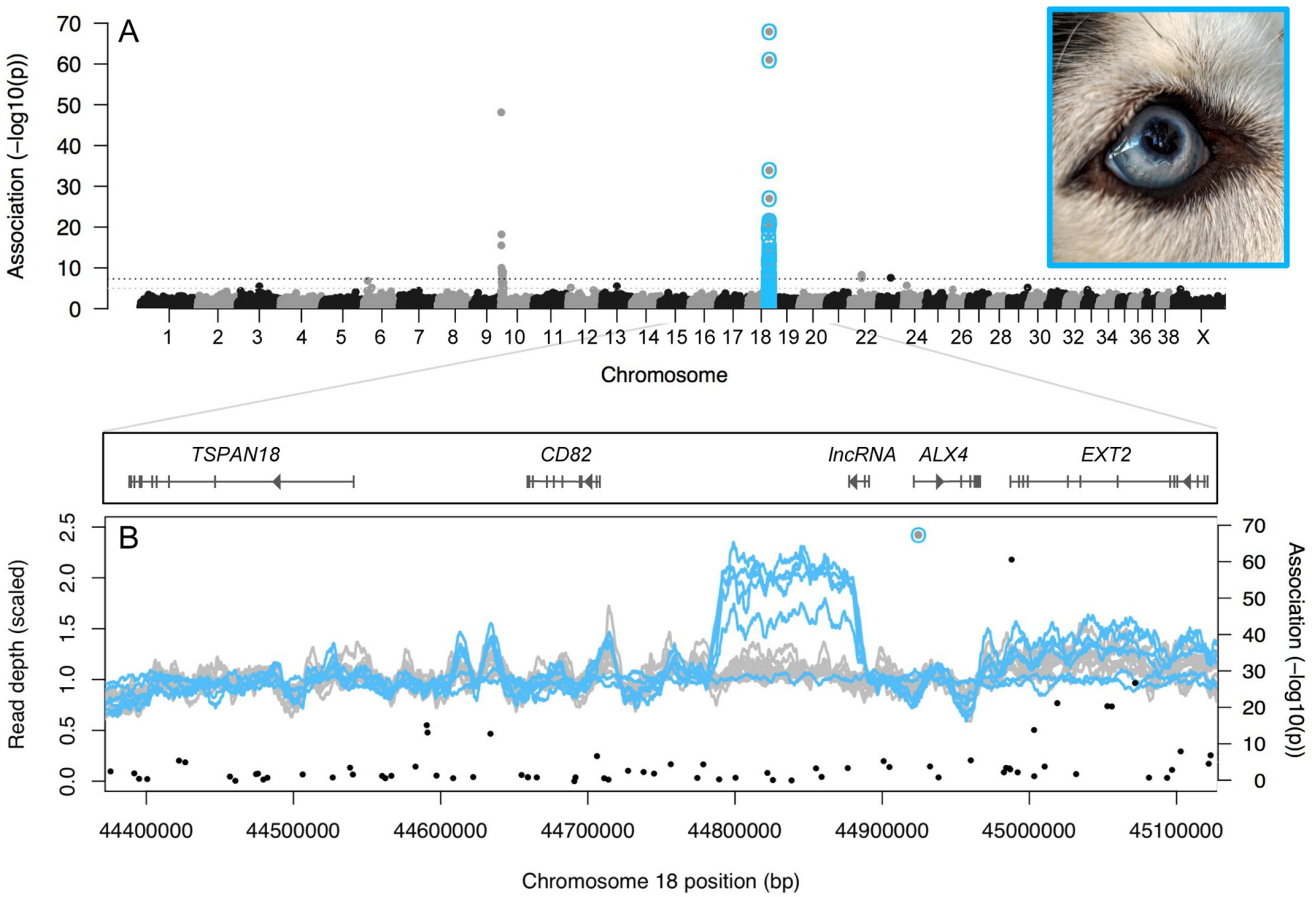
A) Manhattan plot of associations with blue vs. brown eyes across the genomes of 3,180 dogs. Horizontal lines represent the thresholds for suggestive (grey; *P* < 1x10^-5^) and significant (black; *P* < 5x10^-8^) associations. B) Read depth (scaled by the average depth across the interval for each dog) in 10-kb sliding windows across the CFA18 GWAS peak region, for the six Siberian Huskies with publicly available whole genome sequence data (blue) and 11 dogs from other breeds (grey). Five of the six huskies and five of the 11 other breeds carry the GWAS allele associated with blue eyes (dot at 44,924,848). Black vertical lines indicate paired-end reads that aligned 98.6-kb from their mate and in an opposite orientation. Photo credit: Aleksey Gnilenkov (Flickr).

Using a discovery panel of 3,180 dogs, we performed a genome-wide association study and detected two significant associations with blue eyes, one on chromosome 10 at *PMEL17* (“merle”; canFam3.1 position 292,851; *P* = 7.5x10^-49^) and a novel locus on chromosome 18 (CFA18) that had not been previously characterized (position 44,924,848; *P* = 1.3x10^-68^; Fig 1A, S1 Fig). Markers near *MITF* were not significantly associated with blue eyes (*P* = 0.02–0.90 from positions 21,834,567–21,848,176 on CFA20), likely because piebald coat color causes blue eyes in only a small subset of cases.

The novel association on CFA18, located in the first intron of *ALX4*, was robust to whether heterochromia (complete or sectoral) was considered (solid blue only *P* = 3x10^-71^, heterochromia only *P* = 1x10^-12^; S2 Fig), and remained strong when we restricted our analysis to only purebred or mixed-breed dogs (purebred *P* = 3x10^-9^, mixed-breed *P* = 3x10^-63^; S3 Fig). Although the minor allele (A) at the CFA18 locus was carried (in one or two copies) by only 10% of dogs in this dataset (both blue- and brown-eyed), it was carried by 78% of non-merle blue-eyed dogs (32% homozygous, 68% heterozygous) and 100% of blue-eyed purebred Siberian Huskies (*N* = 22).

### Identification and characterization of a 98.6 kilobase duplication linked to the associated allele in *ALX4*

Supplemental Illumina microarray data, specifically log-transformed probe intensity data (*log R*), were available for 87% of the discovery panel dogs (*N* = 2,769 total, *N* = 108 blue-eyed dogs) and from these we defined a fine-mapping panel using 314 dogs that did not carry merle and carried at least one copy of the CFA18 allele associated with blue eyes. Of these, 87 (26%) had at least one blue eye. All blue-eyed dogs homozygous for the CFA18 marker (*N* = 26) shared a long haplotype in the region containing that SNP (S4 Fig; positions 44,633,453–45,170,144), and 92% were homozygous for that haplotype (*N* = 18) or a core subset of that haplotype from positions 44,737,897–45,170,144 (*N* = 6). Within this core haplotype, however, we observed four SNPs (positions 44,800,358, 44,822,014, 44,825,760 and 44,849,276) that were frequently heterozygous, suggesting a non-balanced structural variant overlapping those markers in dogs carrying the blue-eyed haplotype (S4 Fig).

To examine this putative structural variant, we used 17 canine whole genome sequences available on the NCBI Sequence Read Archive (SRA). These sequences included five Siberian Huskies and five representatives of other breeds that were heterozygous or homozygous for the CFA18 allele associated with blue eyes (S1 Table). Genome-wide read depth for the Siberian Huskies carrying one or two copies of the allele abruptly increased across an intergenic region from 44.79–44.89-Mb that encompasses the four frequently heterozygous SNPs in our microarray data (Fig 1B; S1 Table). Furthermore, 30% of the paired-end reads spanning 44,791,417–44,791,584 had a mate that mapped in an opposite orientation to positions 44,890,024–44,890,166 (S5 Fig), consistent with a 98.6-kb tandem duplication for which the midpoint span was less than the insert size of the paired end reads (< 350-bp) [20, 21]. Increased read depth and evidence of a duplication from paired-end mapping was not observed in a sixth Siberian Husky that did not carry the CFA18 allele associated with blue eyes, nor was it observed in other breeds related to Siberian Huskies for which whole genome sequences were available through SRA (e.g. East Siberian Laika, Alaskan Malamute, Samoyed, German Shepherd Dog; Fig 1B).

In the resequenced data, the haplotype bearing the associated CFA18 allele, and the 98.6-kb duplication identified from read depth and paired-end read orientation, contained 48 other variants within a 1Mb window that were not seen on other haplotypes (28 SNPs, and 19 indels that ranged from 1-bp to 30-bp in length; S2 Table). Only two of these candidate SNPs occurred in coding regions (at 45,253,714 and 45,253,740 in the first exon of *CHID1*), and both were synonymous changes. Two small insertions occurred at 44,963,936 in *ALX4* and at 45,140,589 in an *ACS* homolog, but the variants fell in the 3’ untranslated regions (UTRs) of both genes. We therefore prioritized the duplication for further investigation as it was most likely to be the causal variant underlying the phenotype, or very closely associated with the causal variant.

**Fig 2:**
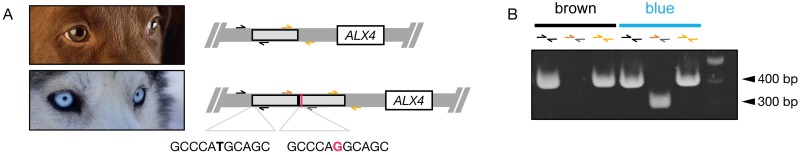
PCR genotyping of a tandem duplication upstream of *ALX4* associated with blue eye color. A)* Schematic view of brown- and blue-eyed alleles (not to scale). The duplication sits head to tail to the ancestral sequence. Three sets of primers were used to amplify three regions (primers denoted with single headed arrows). Sanger sequencing of the duplication midpoint show nearly perfect synteny to canFam3.1 chr18:44791409–44791553 and 44890066–44890185. A single basepair difference, highlighted in red, show a T in the duplication sequence that corresponds to a G at chr18:44791413 in the ancestral sequence. B) PCR genotyping of one brown-eyed and one blue-eyed dog. Primer pairs denoted above each PCR lane. The 5' and 3' flanking regions amplify in both the brown- and blue-eyed alleles; the duplication midpoint amplifies only in the blue-eyed allele.

To characterize the duplication, we designed forward and reverse PCR primers to amplify the midpoint span of the duplication (mapping to CanFam3.1 chr18: 44,890,025–44,890,047 and chr18: 44,791,538–44,791,564 respectively), as well as the 5’ and 3’ ends of the duplicated region as controls (Fig 2; S3A Table). Midpoint products amplified from three blue-eyed purebred huskies, and three mixed-breed dogs with Siberian Husky ancestry: one blue-eyed, predicted to carry the duplication based on log R data, and two brown-eyed, also predicted to carry the duplication based on log R data but with German Shepherd Dog ancestry. Sequencing for all midpoint products in all dogs were identical (S3B Table) and were approximately 300-bp in size. This was consistent with a tandem or near-tandem duplication, which we inferred based on 118-bp of sequence between our forward primer and midpoint, 123-bp of sequence between the reverse primer and midpoint break, and 50-bp of the forward and reverse primers themselves, leading to an 289-bp product in the event of a clean tandem duplication. The sequence aligned with greater than 98% homology to CanFam3.1 chr18: 44,791,409–44791566 and chr18: 44,890,025–44,890,185, as predicted.

### Linkage- and microarray intensity-based inference of associated duplication

**Fig 3:**
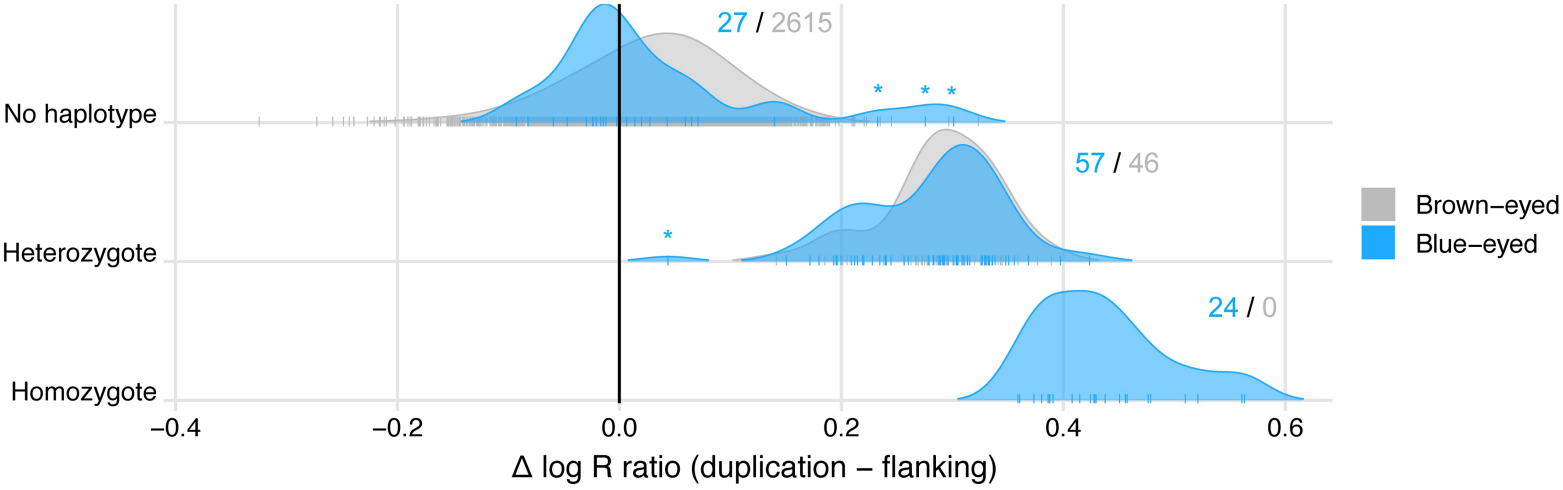
Scaled density plot of Δ *log R* distributions for discovery panel dogs with zero, one, or two copies of the associated haplotype, demonstrating that the presence of the haplotype tracks the presence of the duplication in almost all cases. Dogs carrying the haplotype exhibited elevated *log R* at SNPs within the duplicated region compared to flanking regions (high Δ *log R*) relative to non-carriers, and dogs heterozygous vs. homozygous for the haplotype exhibited distinct distributions, consistent with being heterozygous vs. homozygous for the duplication itself. Although the duplication appeared to act dominantly in Siberian Huskies, brown-eyed heterozygotes in other breeds or mixed breed dogs also had *log R* data consistent with carrying the duplication. Exceptions included three high-*log R* dogs with alternative, recombinant versions of the associated haplotype (asterisks, top panel; S7 Fig) and one low-*log R* dog with a partial duplication (asterisk, middle panel; Supplementary Information). Individual *log R* values contributing to each density curve are represented with vertical ticks, and counts of blue-eyed vs. brown-eyed dogs are indicated for each haplotype category.

We tested for the presence of the core haplotype associated with the blue-eyed phenotype (*N* = 43 markers, excluding those located within the duplication; S4 Fig), and compared *log R* for SNPs located inside vs. outside the duplicated region (Δ *log R*) for dogs that did not carry the haplotype, or were heterozygous or homozygous (Fig 3).

The presence of the duplication-associated haplotype (in one or two copies) explained 75% of blue-eyed cases (*N* = 81 / 108) and was rare in brown-eyed dogs (*N* = 46 / 2,661). Indeed, the haplotype bearing the duplication predicts the blue-eyed phenotype considerably better than the most associated SNP in our GWAS analysis (chi-square duplication *P* = 5.2x10^-290^; GWAS SNP *P* = 4.9x10^-120^; S4 Table). Atypical coat pigmentation or facial markings explained the remaining 25% of cases (Supplementary Information), with the exception of three blue-eyed mixed-breed dogs that possessed recombinant versions of the core haplotype (S7 Fig).

Heterozygotes and homozygotes exhibited distinct distributions of Δ *log R* (*P* = 2.0x10^-13^) consistent with the haplotype also carrying the duplication in these breeds (Fig 3; S8 Fig), with the exception of one blue-eyed mixed-breed dog with low Δ *log R* that exhibited *log R* values at individual SNPs suggestive of a partial duplication (Supplemental Information). Dogs that did not carry the associated haplotype had similar *log R* intensity at SNPs within the duplicated region compared to flanking regions, with a lower Δ *log R* distribution that overlapped with zero (*P* = 2.2x10^-16^ comparing dogs without the haplotype to those with it), indicating that they did not possess the duplication (with the exception of the three recombinant haplotypes discussed above).

### The duplication upstream of *ALX4* remains strongly associated with blue-eyed phenotype in a 2,890 dog validation panel

We compiled a dataset of 2,890 diverse dogs distinct from those included in our GWAS panel to perform a validation test of the association between the duplication and blue eyes. The haplotype existed at low frequency in this panel (41 homozygotes, 26 heterozygotes) and all but two carriers had Δ *log R* values above the minimum bounds observed for heterozygotes in the discovery panel (*N* = 67 / 2,890 with Δ *log R* > 0.15), indicating that the duplication was almost always present on this haplotype. Most dogs that possessed the haplotype and the duplication were Siberian Huskies (*N* = 59 / 67; 41 homozygotes, 17 heterozygotes; S9 Fig), and the remainder were Klee Kai (a breed derived from Siberian Husky; *N* = 2), Australian Shepherd (*N* = 5), and one Australian Cattle Dog (S10 Fig; S5 Table). Profile photos were available for 67% of dogs with the haplotype and duplication (*N* = 46 / 68), and all but one had blue or heterochromic eyes instead of solid brown. The exception was a Siberian Husky with brown eyes despite having one copy of the haplotype (S9 Fig) and Δ *log R* values consistent with being heterozygous for the duplication on that haplotype (0.31). The owner/breeder of this dog was able to provide additional information that confirmed it was a likely carrier of the duplication: It had blue-eyed parents and had sired all blue-eyed or heterochromic litters. The two haplotype carriers with low *log R* (suggesting that the duplication was not present on the haplotype in their case) were both Australian Shepherds, one brown-eyed and one with unknown eye color (no profile photo available).

## Discussion

In this study, we discovered a haplotype containing a 98.6-kb duplication that is strongly predictive of blue eyes and heterochromia in dogs. While we cannot definitively rule out a different typed or untyped variant on this haplotype causing the trait, we feel that the duplication is a plausible causal candidate worthy of further functional investigation. We were able to validate the presence of this duplication with three independent methods: log R intensity from our microarray data, PCR and Sanger sequencing, and read-depth analysis of 17 whole-genome sequences. Further, we showed strong concordance between log R intensity and a consistent haplotype identified from blue-eyed cases in phased haplotype data. Using those phased haplotypes, we found that the duplication-carrying haplotype was more strongly associated with the blue-eyed phenotype than any single marker on our genotyping array (chi-square duplication *P* = 5.2x10^-290^; GWAS SNP *P* = 4.9x10^-120^; S4 Table). Further investigation of variant calls in resequencing data, a comparison of sequences between carriers and non-carriers of the duplication, interestingly revealed no convincing functional targets (S2 Table). While direct functional validation of the duplication is outside of the scope of this research, we suggest that the proximity of this duplication to *ALX4* makes it a prime candidate for functional investigation.

To date, the most familiar examples of duplications affecting phenotype are those related to dosage, cases where one or more duplication events increased gene copy number and, therefore, the amount of translated protein product available for cellular function [22, 23]. However, this duplication sits in an intergenic region between the tetraspanin *CD82* and Homeobox gene *ALX4* (NCBI; UCSC Genome Browser [24]). Two non-coding RNAs (ncRNAs) are annotated on the complementary strand, including an uncharacterized long noncoding RNA (lncRNA) that overlaps the 3’ breakpoint of the duplication (Fig 1; S4 and S6 Figs). We could find no evidence that *CD82* is functionally associated with eye color in humans or any other animal, but *ALX4* and its paralogs play an important role in both mammalian eye development [25, 26] and pigmentation [27].

Research on the genetics of striping patterns in African striped mouse (*Rhabdomys pumilio*) and Eastern chipmunk (*Tamias striatus*) demonstrated that a close paralog of canine *ALX4*, *ALX3*, is a repressor of *MITF* with dorsally striped expression, leading to reduced melanin content and lighter coat color where *ALX3* is upregulated [27]. Gene expression studies in humans have additionally demonstrated that *ALX4* itself is expressed in the RPE [28], and in zebrafish (*Danio rerio*), expression of *ALX4* orthologs, *alx4a* and *alx4b*, are enriched in iridophores, which originate in common with melanocytes from the neural crest [29]. Given the importance of *cis*-regulatory elements in local gene regulation [30, 31] and the location of the duplication upstream of *ALX4*, we propose that this large duplication in a candidate regulatory region could cause blue eyes by increasing expression of *ALX4* in the RPE, leading to repression of *MITF* and a reduction in melanin in the iris.

The high proportion of blue-eyed heterozygotes in our analyses (53% of blue-eyed dogs) suggests that the duplication, if causal, is dominant in its phenotypic effect. However, the existence of 46 brown-eyed heterozygotes with similarly elevated Δ *log R* (*P* = 0.35 comparing blue and brown heterozygote distributions) suggests that one or more additional genetic factors may modify or mask the duplication’s effect on eye color (Fig 3; S8 Fig). This effect is not completely explained by an individual’s genotype at four previously characterized pigmentation genes (A, E, B, and K loci; S6 Table), although carriers of the duplication that also carry at least one copy of the dominant melanistic mask (Em) allele are significantly more likely to have brown eyes than duplication carriers without melanistic mask (P = 0.0018).

Functional follow-up studies are needed to explicitly assay regulatory changes in *ALX4* caused by this duplication; however, we have shown that this mutation is highly (but not completely) penetrant and most common in Siberian Huskies. The presence of the duplication explains at least 75% of blue-eyed cases in our discovery panel of customer dogs (81 / 108 blue-eyed dogs carried the associated haplotype and have elevated Δ *log R* values consistent with duplicated markers).

In summary, by using consumer genomic data to drive this research, we were able to build the largest canine GWAS dataset to date, determine the prevalence of a putatively causal variant, a duplication upstream of *ALX4* highly associated with blue eyes, across a diverse population, and utilize our relationship with owners of specific dogs to learn more about the inheritance of the trait. As more canine genetic testing is done on high-density array platforms, these databases hold particular promise for unlocking the genetic basis of complex phenotypes for which dogs are a particularly useful model, including cancer, behavior, and aging.

## Methods

### Discovery samples

We solicited phenotype data from customers whose dogs have been genetically tested by Embark Veterinary, and who have agreed to participate in research, by implementing an online survey about their dog’s morphological traits at http://embarkvet.com and encouraging participation via email. We initiated the survey on February 7, 2017, and, as of November 23, 2017, owners of 3,248 adolescent and adult dogs whose eye color can be assumed to be developmentally complete (6 months or older) had submitted a response to the section of that survey that asks about eye color (S11 Fig). Most were owners of mixed-breed dogs, and 21% were owners of purebred dogs (*N* = 668 / 3,180). A subset of owners (*N* = 68) selected "other", indicating that their dog had an eye color not represented by any of the seven options. In total, 156 dogs in this dataset were reported to have either solid blue eyes (*N* = 73) or heterochromic eyes (partially blue; *N* = 83), compared to 3,024 with some shade of solid brown. We encoded this trait as a binary phenotype in case-control format (0: brown, 1: blue) and considered both solid blue and heterochromic dogs as cases. Ancestry from 185 different dog breeds, landraces (village dogs) and gray wolves was represented in this dataset.

### Genotyping & quality control

Customer dogs were genotyped on Embark’s custom high-density 214,661-marker platform (213,245 filtered to autosomal, chromosome X, and pseudoautosomal region markers). Total genotyping rate was 99.5%, and all dogs (*N* = 3,180) had less than 2.5% missing data and passed standard filtering in PLINK [32]. After filtering, 90% of variant sites (192,108 / 213,245) were genotyped across at least 95% of individuals and were included in subsequent analyses.

### Genome-wide association

We constructed a relatedness matrix from centered genotypes and ran a genome-wide association test based on a univariate linear mixed-model in GEMMA [33], using the eigenvalues and eigenvectors of the relatedness matrix to control for confounding effects of shared ancestry, particularly among dogs of the same breed or breed group (groups of closely related or recently derived breeds). We identified significant associations by applying a threshold of *P* < 5.0 x 10^−8^ to the Wald test statistic.

### Whole genome sequence analysis

We downloaded whole genome sequence data for 17 dogs from the NCBI Sequence Read Archive [34], conducted an end-to-end alignment using Bowtie2 in—very-sensitive alignment mode [35] calculated read depth coverage across sites using SAMtools [36], and investigated mapped paired-end reads in regions of interest using the Integrative Genomics Viewer (IGV) [37, 38]. For each dog, we calculated the change in read depth between the putative duplicated region, demarcated by discordantly mapping paired-end reads (44791417 to 44890166-bp), and 5Mb of flanking sequence immediately surrounding it (chr18:42999825-44791417 and chr18:44890166-48000173). We called variants across a 1-Mb region surrounding the most associated GWAS SNP using HaplotypeCaller from the Genome Analysis Tool Kit (GATK) [39] for 9 SRA samples (ERR911199, ERR911200, ERR1014362, ERR1990016, SRR1122359, SRR1124049, SRR1124304, SRR1784129, SRR2095539) with robust read depth and compatible alignment formatting (including four huskies with the duplication, and one husky and four other breeds without the duplication). Following batch variant-calling for SNPs and indels independently, we refined the call set by filtering variants that did not meet QC requirements for minimum read depth (SNPs and indels < 2), the phred-scaled p-value from a Fisher’s exact test for strand bias (SNPs > 60, indels > 200), the variant position relative to the end of the read (Mann-Whitney rank sum test, SNPs < -8, indels < -20) and, for SNPs, mapping quality (root mean square < 40, Mann-Whitney rank sum test < -12.5). We then identified 47 SNPs or indels present in the dogs with the duplication, and absent from those without the duplication (S2 Table). Since eye color phenotypes were not available for datasets archived to SRA, this approach was able to identify additional variants that co-segregate with the duplication (and are therefore at least as explanatory for the phenotype), but it was not possible to scan for variants that perform better (i.e. those that might additionally explain brown-eyed duplication carriers).

### PCR amplification of the duplication

We designed primers to amplify the midpoint span of the duplication, as well as the 5’ and 3’ flanking regions of the duplicated region as positive controls. Genomic DNA (gDNA) remaining from microarray analysis (2 uL) was used for PCR reactions using the following primer combinations: ALX4_5Fl_1F + ALX4_5Fl_1R; ALX4_Dup_2F + ALX4_Dup_2R; ALX4_3Fl_1R + ALX4_3Fl_1R. All PCR reactions were performed using Q5 High Fidelity Taq Polymerase (NEB Cat No M0491) in a total volume of 20 uL following the manufacturer’s protocol. The following cycling parameters were used: 98°C 30s, 40X (98°C 10s, 60°C 30s, 72°C 30s), 72°C 5m, 16°C hold. PCR product was visualized on a 1% agarose gel with 1X GelRed (Biotium Cat No 41003); product from 9 dogs were submitted for purification and Sanger sequencing at Genewiz (Genewiz.com).

### Haplotype analysis

Genotype data for all dogs was phased against a proprietary reference panel, with missing data imputed using Eagle2 [40]. We examined phased data around the putative duplication breakpoints in 26 blue-eyed dogs in the GWAS data set that were homozygous for the *CFA18* A allele. This revealed a single haplotype bearing this allele that spanned 81 markers between positions 44,336,453 and 45,170,144 that was present in all 26 dogs in at least one copy (S4 Fig). We further defined a 43 marker core haplotype between positions 44,737,897 and 45,170,144 that was present in two copies in 24 of the 26 dogs, and in one copy in the remaining two dogs. We compared the change in intensity between duplication markers and flanking regions (Δ *log R*) according to both the eye color phenotype and haplotype state of each dog. For all remaining dogs in the discovery panel and all dogs in the validation panel, we calculated the number of copies of the core haplotype present in their phased data (excluding the markers inside the duplication: 44,825,760, 44,838,433, 44,849,276, 44,855,038, 44,858,831, 44,876,627).

### Validation samples

The validation panel included 2,890 dogs of various breeds, none of whom carried the most associated *CFA10* marker for merle. Eye color could be scored for these individuals when customers had uploaded high resolution profile photos. Photos were available for 70% of dogs bearing both the associated haplotype and a *log R* signature of the duplication on that haplotype.

### Ethics statement

Owners of participating dogs were part of the Embark Veterinary, Inc. customer base. Owners provided informed consent to use their dogs’ data in scientific research. Owners provided photographs of their dogs and filled out online survey questions concerning their dog’s eye color; no invasive methods for genotype or phenotype collection were used, nor were dogs ever handled by researchers. Owners were given the opportunity to opt out of the study at any time during data collection. The discovery cohort was selected from data available before August 2017; the fine mapping cohort was selected from data available before Dec 2017. All published data have been deidentified of all Personal Information as detailed in Embark’s privacy policy (embarkvet.com/privacy-policy/).

## Supporting information

S1 Fig QQ plot of the association between genotype and blue vs. brown eyes across the genomes of 3,180 purebred and mixed-breed dogs (corresponds to Manhattan plot in Fig 1). (PNG)

S2 Fig Manhattan and QQ plots of genome-wide associations with A) solid blue eyes (73 cases) and B) heterochromia (83 cases) across the genomes of 3,180 purebred and mixed breed dogs (192,550 markers). Grey and black dotted horizontal lines represent the thresholds for suggestive (*P* < 1x10^-5^) and significant (*P* < 5x10^-8^) associations, respectively.
(PNG)

S3 Fig Manhattan and QQ plots of genome-wide associations for A) 2,448 mixed-breed dogs (192,570 markers) and B) 670 purebred dogs (191,854 markers). Grey and black dotted horizontal lines represent the thresholds for suggestive (*P* < 1x10^-5^) and significant (*P* < 5x10^-8^) associations, respectively.
(PNG)

S4 Fig Structure of a linked haplotype block (core haplotype outlined in black) shared by dogs homozygous for the CFA18 allele associated with blue eyes (marker position indicated by an arrow), featuring four frequently heterozygous markers (asterisks) suggestive of a non-balanced structural variant in the region between the vertical white lines. Each row shows the nucleotide sequence of one haplotype at all markers, and haplotype pairs for each dog are separated by horizontal white lines.
(PNG)

S5 Fig In each Siberian Husky for which read depth increased 1.5-2X across an intergenic region from 44.79–44.89-Mb that encompassed the four heterozygous SNPs (Fig 1B), a subset of reads at positions 44,791,417–44,791,584 had a mate that mapped in an opposite orientation to positions 44,890,024–44,890,166, consistent with a 98.6-kb tandem duplication for which the midpoint span was less than the insert size of the paired end reads (< 350-bp). Visualizations generated via IGV 2.4.10 (Robinson et al. 2011; Thorvaldsdóttir et al. 2013).
(PNG)

S6 Fig Structural diagram for snRNA located within duplicated region (UCSC Genome Browser). (PNG)

S7 Fig Matches (blue) and mismatches (black) to the core haplotype (black box) carrying the CFA18 GWAS allele associated with blue eyes (marker position indicated by an arrow), among blue-eyed dogs without the core haplotype. The vertical white line separates positions upstream and downstream of the duplicated region (markers on the array within the duplicated region are excluded). The majority of blue-eyed dogs included in the GWAS analysis that did not possess the associated haplotype (*N* = 27), were piebald, albino, or had white facial markings overlapping the eyes. However, three blue-eyed dogs with husky ancestry were heterozygous for partial copies of the core haplotype.
(PNG)

S8 Fig Matches (blue) and mismatches (black) to the core haplotype (black box) carrying the *CFA18* GWAS allele associated with blue eyes (marker position indicated by an arrow), among brown-eyed heterozygotes. The vertical white line separates positions upstream and downstream of the duplicated region (duplicated markers excluded). Among dogs in the GWAS analysis, 45% of all heterozygotes for the associated haplotype were brown-eyed (*N* = 46).
(PNG)

S9 Fig Matches (blue) and mismatches (black) to the core haplotype (black box) carrying the CFA18 GWAS allele associated with blue eyes (marker position indicated by an arrow), among purebred Siberian Huskies. The vertical white line separates positions upstream and downstream of the duplicated region (duplicated markers excluded). Assuming a dominant mode of inheritance, possession of the CFA18 haplotype predicted the blue-eyed phenotype in purebred Siberian Huskies, with one exception (one brown-eyed husky was a heterozygote).
(PNG)

S10 Fig Matches (blue) and mismatches (black) to the core haplotype (black box) carrying the CFA18 GWAS allele associated with blue eyes (marker position indicated by an arrow), among purebred non-huskies from the validation panel that possess the haplotype. The vertical white line separates positions upstream and downstream of the duplicated region (duplicated markers excluded).
(PNG)

S11 Fig Participants were prompted to report their dog’s eye color as one of seven options, guided by visual examples (images courtesy of musingsofabiologistanddoglover.blogspot.com and quaggatale.wordpress.com). (PNG)

S1 Table Canine whole genome sequences available on the NCBI Sequence Read Archive (SRA), used for duplication fine-mapping via paired-end read orientation, a comparative analysis of read depth, and additional variant calling across the region. *indicates Siberian Husky samples carrying the duplication.
(DOCX)

S2 Table 47 variants within 1-Mb of the top GWAS SNP, distinguishing huskies with the duplication from 1 husky and other sequenced breeds available on SRA that do not carry the duplication. (DOCX)

S3 Table (a) Primer sequences used for PCR assays described in Fig 2. (b) Midpoint span product sequence. The T>G SNP that differentiates original and duplicated copies is indicated in bold.
(DOCX)

S4 Table Contingency table comparison for the duplication vs. the top associated GWAS SNP, in our discovery panel of 2,769 mixed-breed and purebred dogs. (DOCX)

S5 Table Frequency of the CFA18 core haplotype bearing a duplication associated with blue eyes in the discovery panel (a), and the validation panel (b). The haplotype was not present in 195 / 201 breeds represented across the discovery and validation panels.
(DOCX)

S6 Table A-locus ASIP (A), E-locus MC1R (B), B-locus TYRP1 (C) and K-locus CBD103 (D) genotype frequencies among blue-eyed (N = 147) and brown-eyed (N = 47) duplication carriers, indicated as the percent of total (% blue / % brown). (DOCX)

S1 Text (DOCX)
