# Supplementary figures and images for: Direct-to-consumer DNA testing of 6,000 dogs reveals 98.6-kb duplication associated with blue eyes and heterochromia in Siberian Huskies
Source: PLoS Genet. 2018 Oct 4;14(10):e1007648. doi: 10.1371/journal.pgen.1007648 (PMC6171790; doi:10.1371/journal.pgen.1007648)

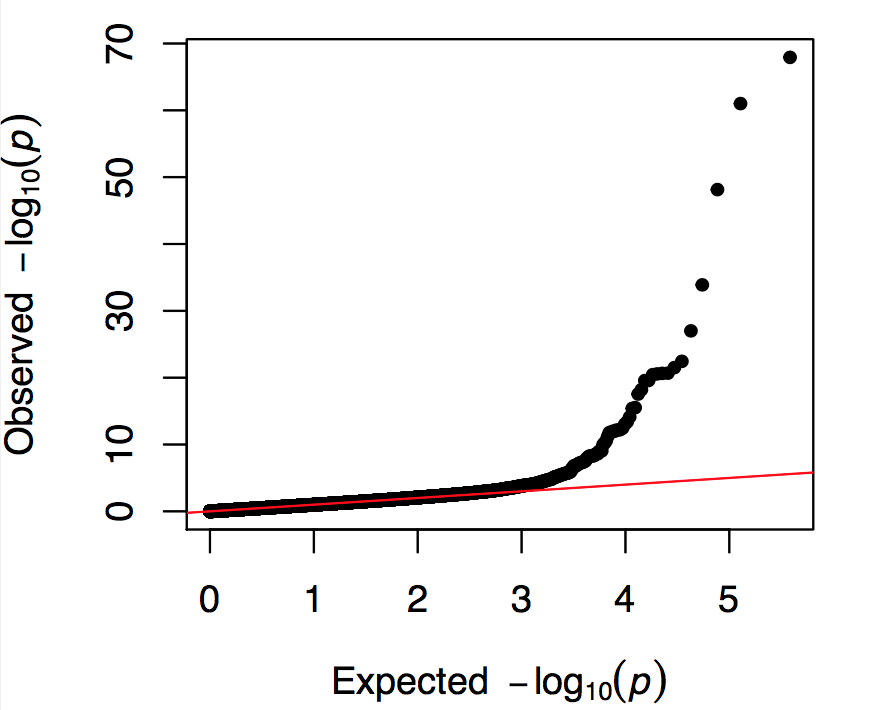

Supplement: S1 Fig — (PNG) [file pgen.1007648.s001.png]

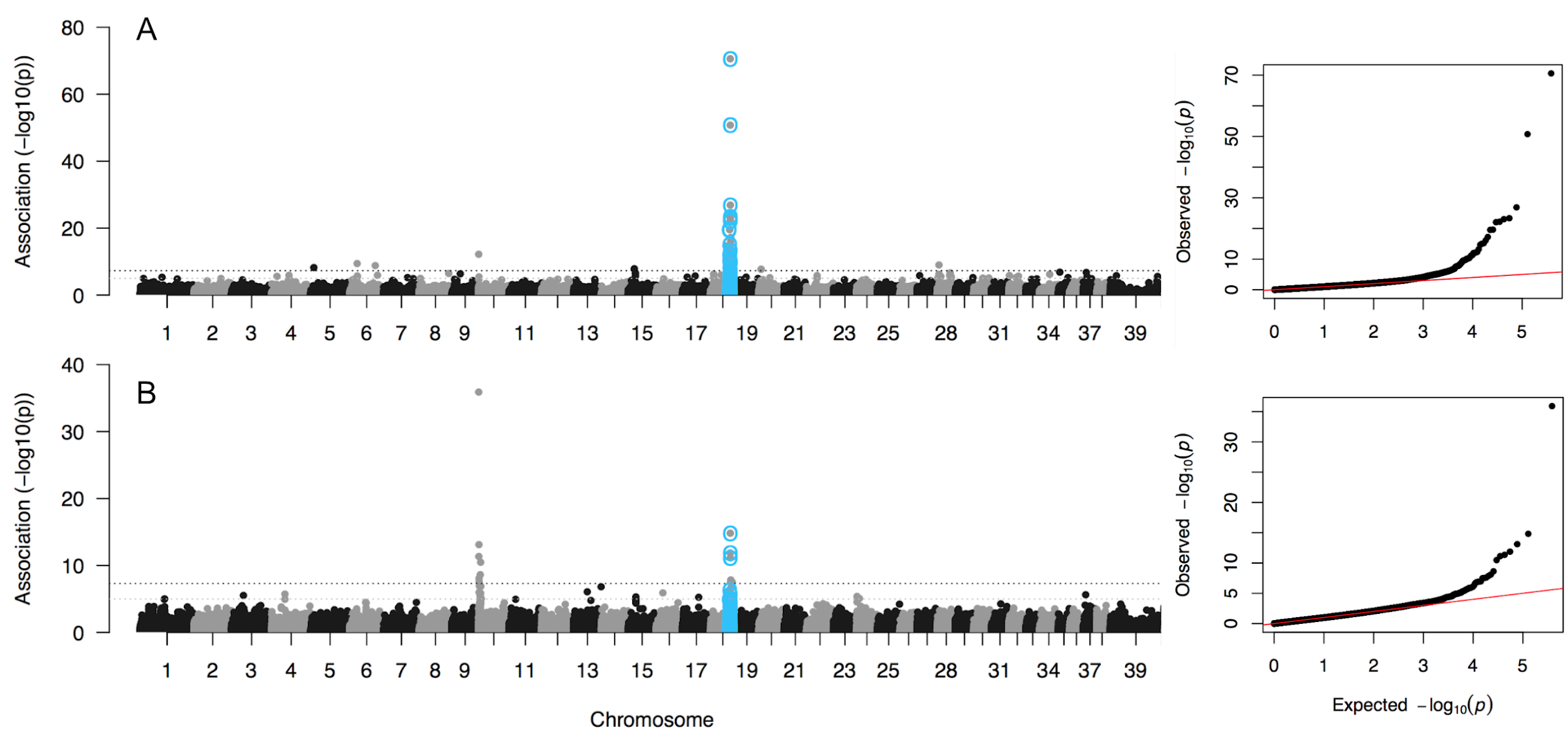

Supplement: S2 Fig — Grey and black dotted horizontal lines represent the thresholds for suggestive (P < 1x10^-5^) and significant (P < 5x10^-8^) associations, respectively. (PNG) [file pgen.1007648.s002.png]

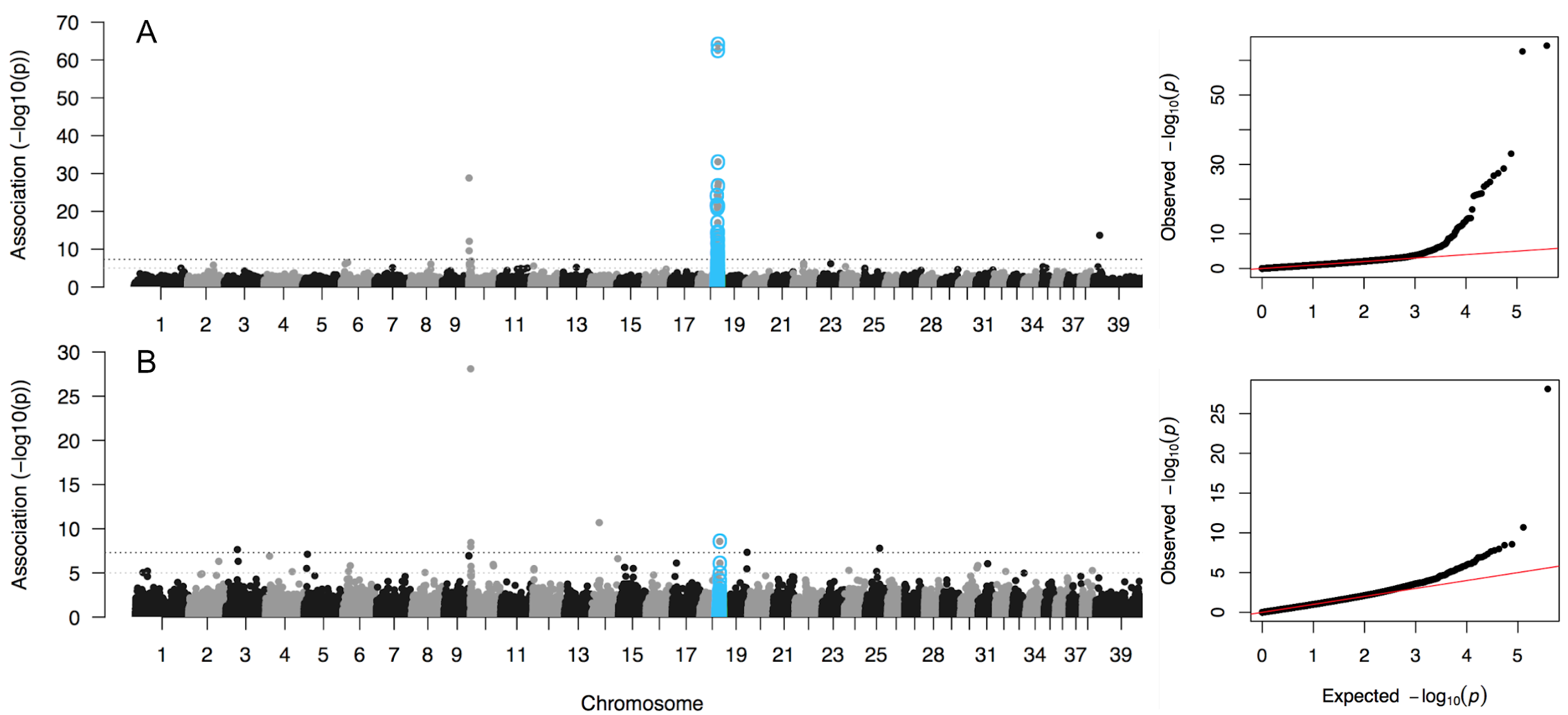

Supplement: S3 Fig — Grey and black dotted horizontal lines represent the thresholds for suggestive (P < 1x10^-5^) and significant (P < 5x10^-8^) associations, respectively. (PNG) [file pgen.1007648.s003.png]

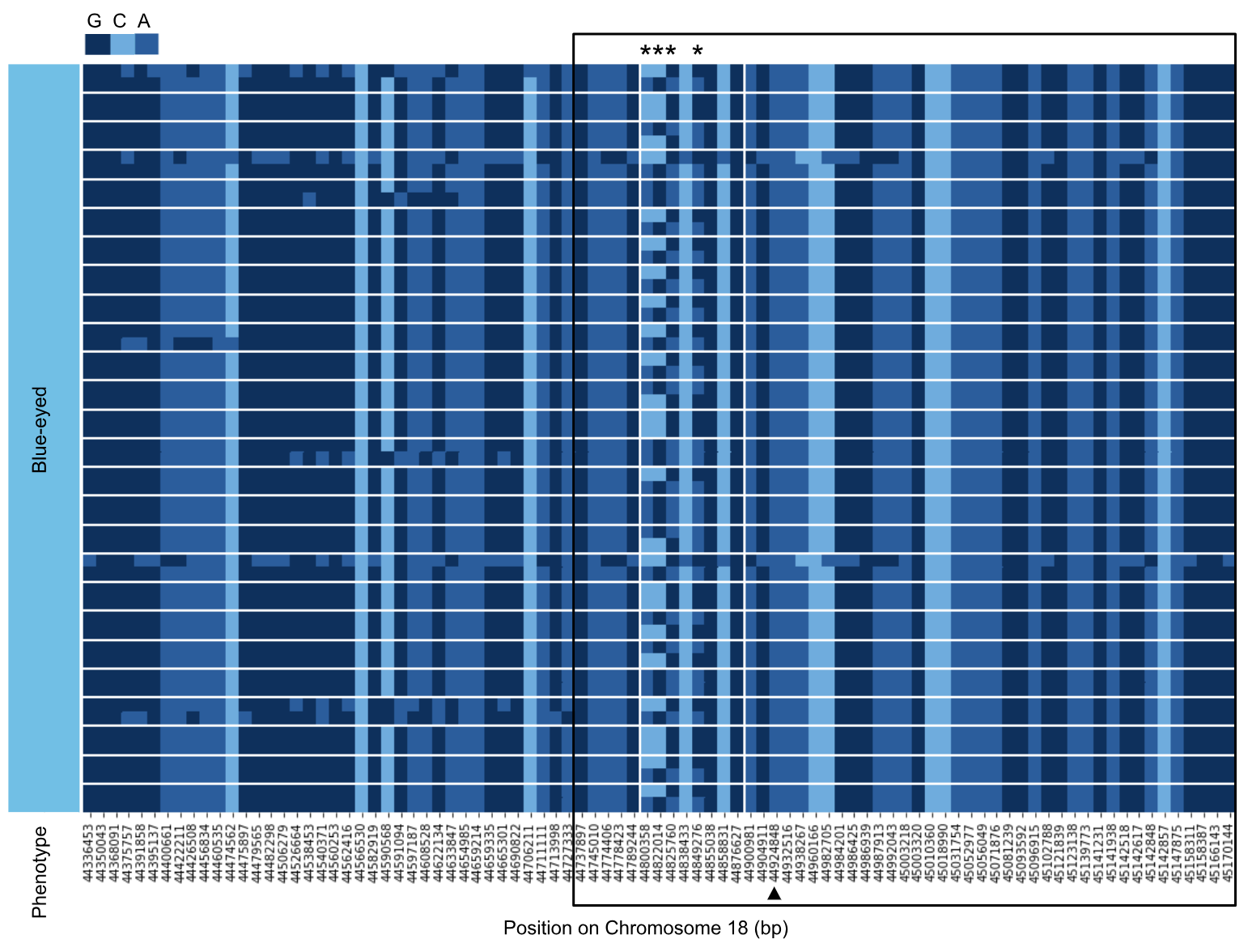

Supplement: S4 Fig — Each row shows the nucleotide sequence of one haplotype at all markers, and haplotype pairs for each dog are separated by horizontal white lines. (PNG) [file pgen.1007648.s004.png]

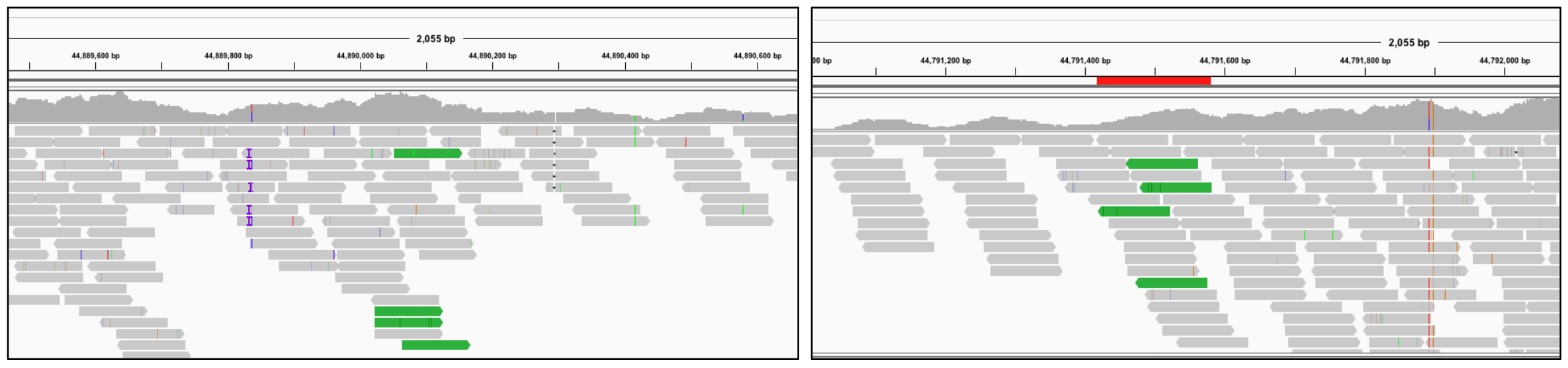

Supplement: S5 Fig — Visualizations generated via IGV 2.4.10 (Robinson et al. 2011; Thorvaldsdóttir et al. 2013). (PNG) [file pgen.1007648.s005.png]

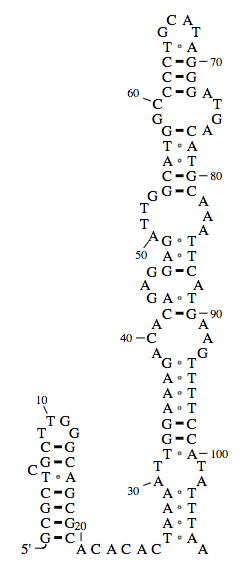

Supplement: S6 Fig — (PNG) [file pgen.1007648.s006.png]

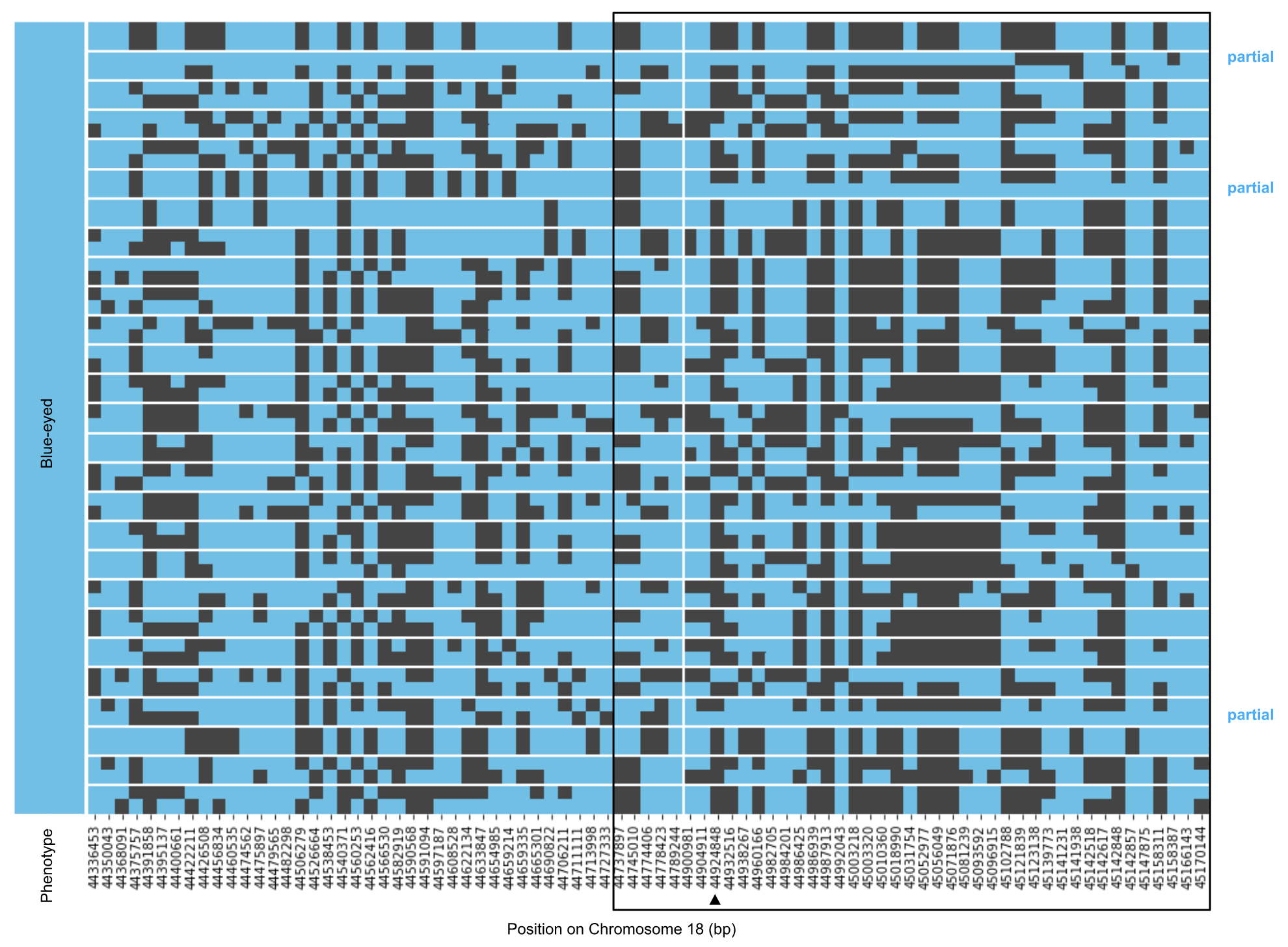

Supplement: S7 Fig — The vertical white line separates positions upstream and downstream of the duplicated region (markers on the array within the duplicated region are excluded). The majority of blue-eyed dogs included in the GWAS analysis that did not possess the associated haplotype (N = 27), were piebald, albino, or had white facial markings overlapping the eyes. However, three blue-eyed dogs with husky ancestry were heterozygous for partial copies of the core haplotype. (PNG) [file pgen.1007648.s007.png]

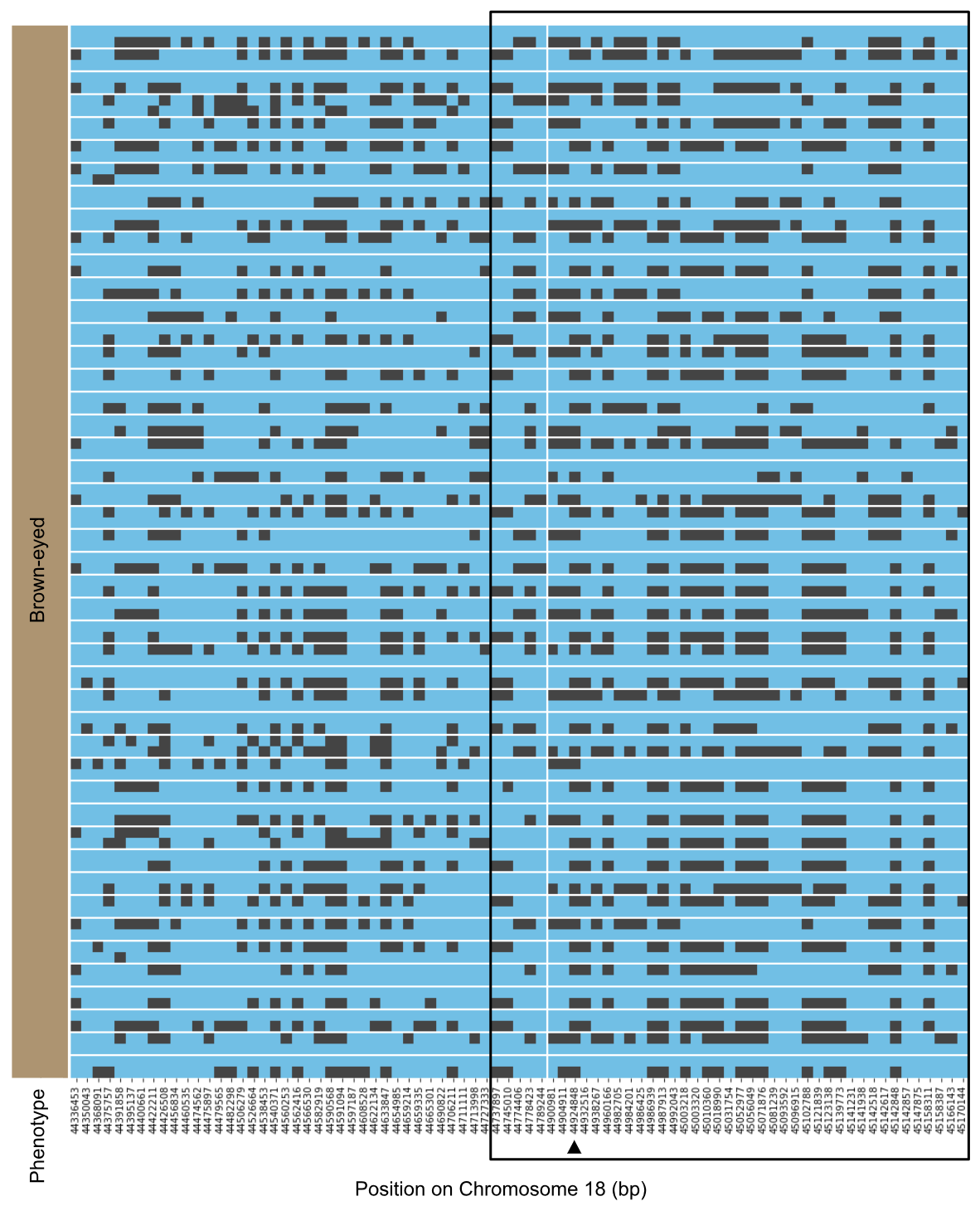

Supplement: S8 Fig — The vertical white line separates positions upstream and downstream of the duplicated region (duplicated markers excluded). Among dogs in the GWAS analysis, 45% of all heterozygotes for the associated haplotype were brown-eyed (N = 46). (PNG) [file pgen.1007648.s008.png]

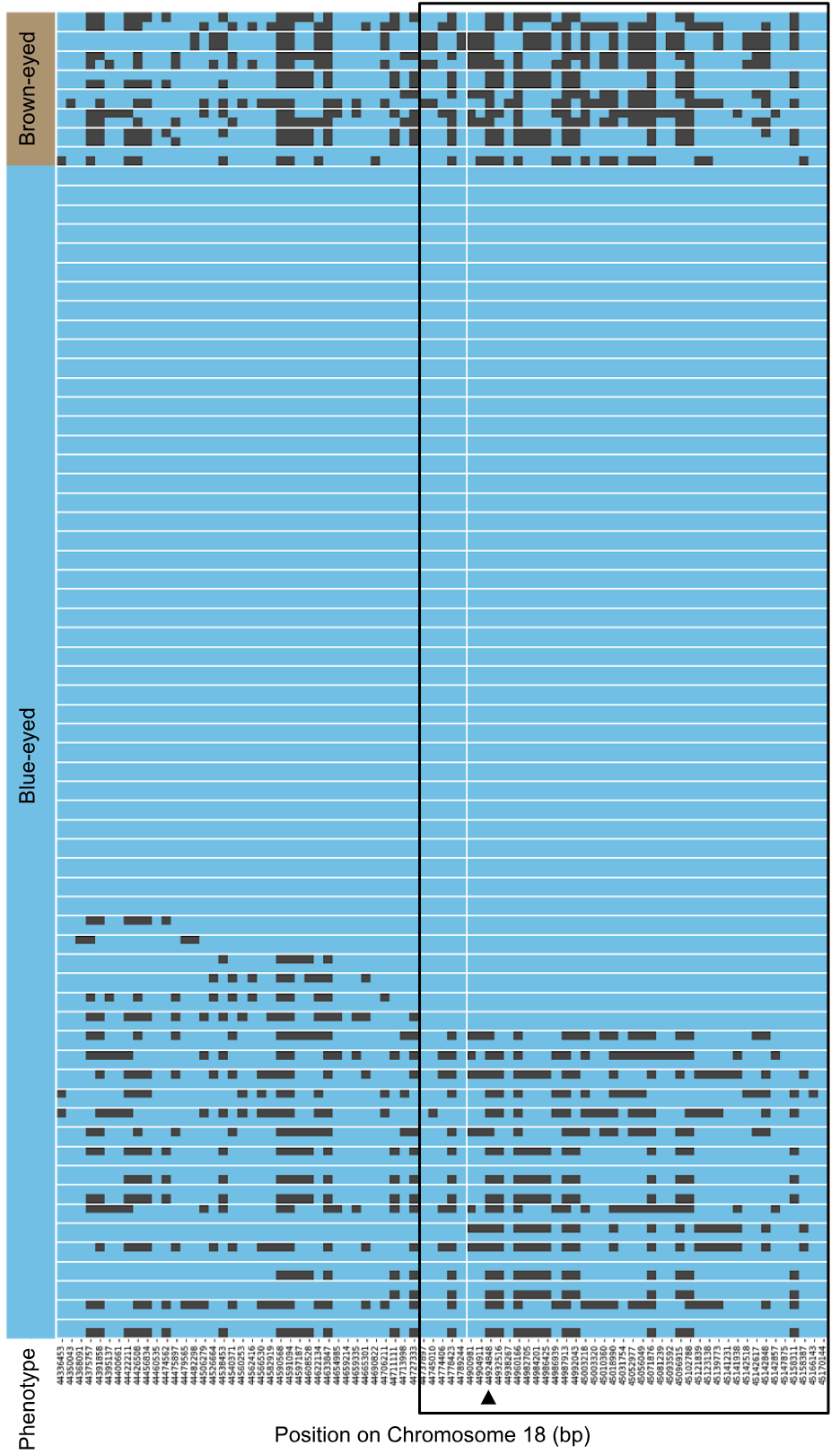

Supplement: S9 Fig — The vertical white line separates positions upstream and downstream of the duplicated region (duplicated markers excluded). Assuming a dominant mode of inheritance, possession of the CFA18 haplotype predicted the blue-eyed phenotype in purebred Siberian Huskies, with one exception (one brown-eyed husky was a heterozygote). (PNG) [file pgen.1007648.s009.png]

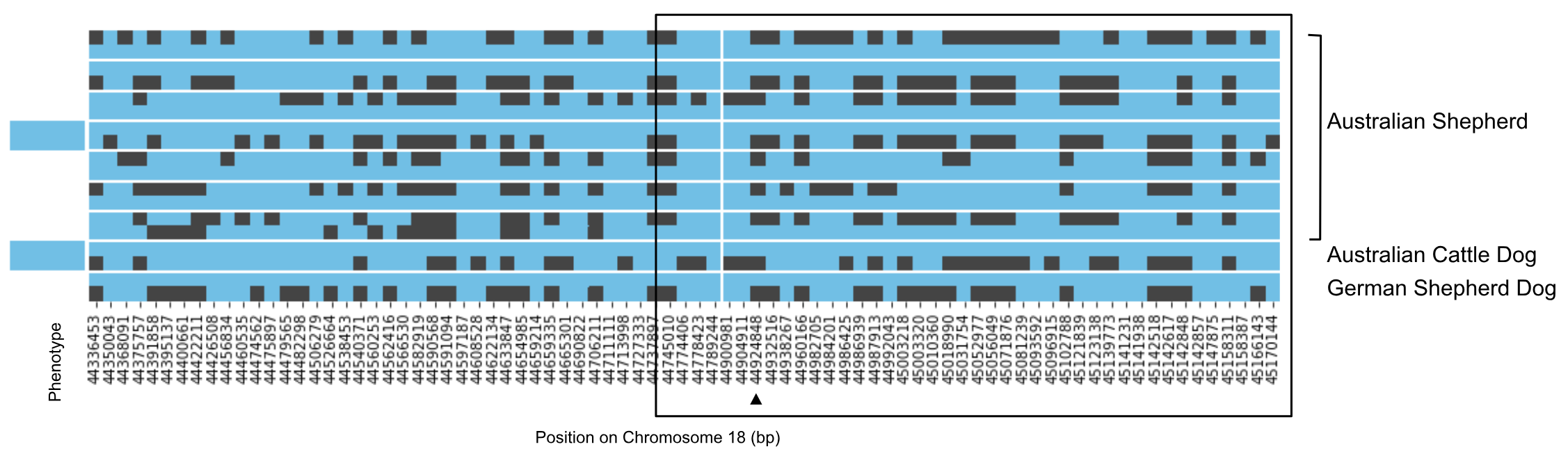

Supplement: S10 Fig — The vertical white line separates positions upstream and downstream of the duplicated region (duplicated markers excluded). (PNG) [file pgen.1007648.s010.png]

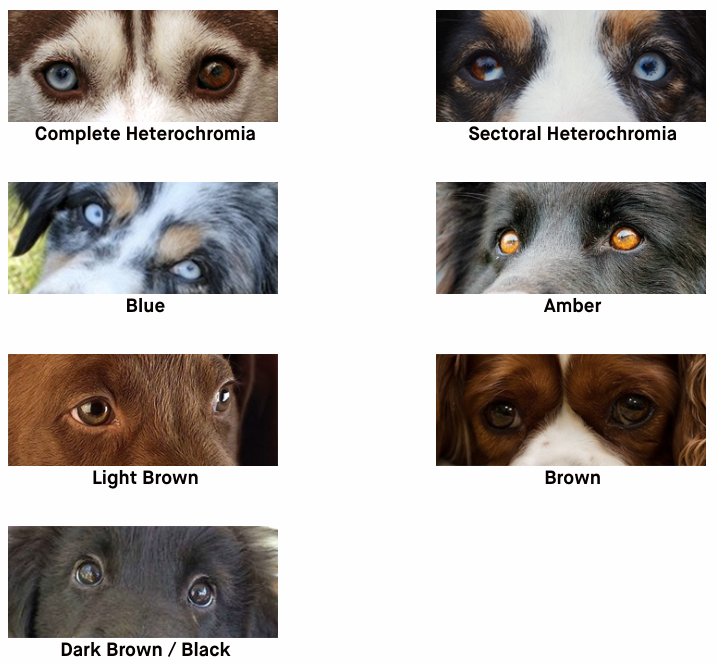

Supplement: S11 Fig — (PNG) [file pgen.1007648.s011.png]
