# Supplementary material for: Direct-to-consumer DNA testing of 6,000 dogs reveals 98.6-kb duplication associated with blue eyes and heterochromia in Siberian Huskies
Source: PLoS Genet. 2018 Oct 4;14(10):e1007648. doi: 10.1371/journal.pgen.1007648 (PMC6171790; doi:10.1371/journal.pgen.1007648)
Supplement: S1 Table — *indicates Siberian Husky samples carrying the duplication. (DOCX) [file pgen.1007648.s012.docx]

| ID | Breed | Δ read depth  (duplication - 5-Mb flanking region) |
| --- | --- | --- |
| ERR911199 | Siberian Husky* | 13.45 |
| ERR911200 | Siberian Husky* | 13.65 |
| ERR1014362 | Siberian Husky* | 7.20 |
| ERR1990016 | Siberian Husky | 0.35 |
| SRR2095539 | Siberian Husky* | 47.14 |
| SRR3384079 | Siberian Husky* | 5.95 |
| SRR1122359 | German Shepherd Dog | 0.63 |
| SRR1124049 | German Shepherd Dog | 0.50 |
| SRR1124304 | German Shepherd Dog | 0.54 |
| SRR4011155 | German Shepherd Dog | 1.05 |
| SRR1784129 | Nova Scotia Duck Tolling Retriever | 0.44 |
| SRR2827563 | Alaskan Malamute | 1.27 |
| SRR2827565 | East Siberian Laika | 1.07 |
| SRR2827573 | Samoyed | 2.13 |
| SRR3384105 | Beagle | 0.24 |
| SRR7107658 | Afghan Hound | 0.41 |
| SRR7107945 | Basenji | 0.72 |
