# Supplementary material for: Direct-to-consumer DNA testing of 6,000 dogs reveals 98.6-kb duplication associated with blue eyes and heterochromia in Siberian Huskies
Source: PLoS Genet. 2018 Oct 4;14(10):e1007648. doi: 10.1371/journal.pgen.1007648 (PMC6171790; doi:10.1371/journal.pgen.1007648)
Supplement: S2 Table — (DOCX) [file pgen.1007648.s013.docx]

| Position | A1/A2 | Position | A1/A2 |
| --- | --- | --- | --- |
| 44462798 | CAAAAAAA/C | 44956150 | C/T |
| 44490149 | TA/T | 44956226 | T/C |
| 44520917 | A/G | 44957940 | C/T |
| 44521368 | C/A | 44958456 | CTG/C |
| 44578831 | G/C | 44963936 | C/CT |
| 44579861 | T/C | 44964070 | A/G |
| 44580325 | G/A | 44976637 | TAGTCTTAAGACTATTAAGACTTAATAGTC/T |
| 44588860 | C/A | 44996123 | CAGCTACCTTAAGAAA/C |
| 44588869 | A/C | 45084270 | A/AAC |
| 44601195 | G/T | 45100645 | C/CT |
| 44607025 | T/C | 45100648 | C/A |
| 44626711 | G/C | 45103069 | G/A |
| 44632293 | T/TC | 45103144 | C/T |
| 44661505 | ACCAGGCGGGCCCACCCCGACTCGGGCCG/A | 45129130 | T/TG |
| 44661508 | G/A | 45140589 | A/AAAAG |
| 44698795 | A/C | 45157128 | AC/A |
| 44706651 | T/G | 45163986 | C/G |
| 44739553 | AC/A | 45167656 | GGGGGA/G |
| 44768955 | C/A | 45248672 | G/A |
| 44874575 | TAAAAATA/T | 45253714 | G/C |
| 44910482 | C/CA | 45253740 | T/C |
| 44922960 | C/T | 45283999 | CTTTTTTTTT/C |
| 44929751 | C/CACA | 45411767 | G/A |
| 44929753 | C/T |  |  |
