# Supplementary material for: Direct-to-consumer DNA testing of 6,000 dogs reveals 98.6-kb duplication associated with blue eyes and heterochromia in Siberian Huskies
Source: PLoS Genet. 2018 Oct 4;14(10):e1007648. doi: 10.1371/journal.pgen.1007648 (PMC6171790; doi:10.1371/journal.pgen.1007648)
Supplement: S3 Table — (a) Primer sequences used for PCR assays described in Fig 2. (b) Midpoint span product sequence. The T>G SNP that differentiates original and duplicated copies is indicated in bold. (DOCX) [file pgen.1007648.s014.docx]

**a.**

| Primer Name | Sequence | Purpose |
| --- | --- | --- |
| ALX4_Dup_2F | GGGAGCAAAAGAAACAATAGGGA | Forward primer for duplication midpoint |
| ALX4_Dup_2R | AGAGACTATTATTGTCTGCATTTTACA | Reverse primer for duplication midpoint |
| ALX4_3Fl_1F | TCAAGTCAGGCCGGAAAGTC | Forward primer for 3’ flanking region |
| ALX4_3Fl_1R | GACCTGCCACTCTTGCTGAT | Reverse primer for 3’ flanking region |
| ALX4_5Fl_1F | GCTCATGGCAGACATTCTCAATC | Forward primer for 5’ flanking region |
| ALX4_5Fl_1R | ACACTGCCAGGACTTTGCTT | Reverse primer for 5’ flanking region |

**b.** TGGGAGCAAAAAGAAACAATAGGGAAAGCTAGAAAGTGTGTGTAGCACAGCCCTGGAAAC TTTATCCTGGCTGGATCCCCCATCCAGGGGGCCCCGCAAGGAGTCCTGGAGCCACCCGGC
AGAGCCAAATGCGGATCAGCAAGAGTGGCAGGTCCCA**G**GCAGCCTGTATAATCCCCAGCA
GAGTAAGAGATGAAAGCAAAACCTTCCAAGAGGAGGGAGCAGAAAGCAGGGGTAAATGTG
AGGGTTGGTGCAGACATGAATCCAAAATCAGCCTCTGCTGATTGAAAAATGCAGACAATA
ATAGTCTCTAA
