# Supplementary material for: Direct-to-consumer DNA testing of 6,000 dogs reveals 98.6-kb duplication associated with blue eyes and heterochromia in Siberian Huskies
Source: PLoS Genet. 2018 Oct 4;14(10):e1007648. doi: 10.1371/journal.pgen.1007648 (PMC6171790; doi:10.1371/journal.pgen.1007648)
Supplement: S4 Table — (DOCX) [file pgen.1007648.s015.docx]

|  | Marker absent | Marker present |
| --- | --- | --- |
| Blue | Duplication: 24  GWAS allele: 20 | Duplication: 84  GWAS allele: 88 |
| Brown | Duplication: 2,615  GWAS allele: 2,435 | Duplication: 46  GWAS allele: 226 |
