# Supplementary material for: Direct-to-consumer DNA testing of 6,000 dogs reveals 98.6-kb duplication associated with blue eyes and heterochromia in Siberian Huskies
Source: PLoS Genet. 2018 Oct 4;14(10):e1007648. doi: 10.1371/journal.pgen.1007648 (PMC6171790; doi:10.1371/journal.pgen.1007648)
Supplement: S5 Table — The haplotype was not present in 195 / 201 breeds represented across the discovery and validation panels. (DOCX) [file pgen.1007648.s016.docx]

**a.**

| Breed | N | No haplotype | Heterozygote | Homozygote | Haplotype freq. |
| --- | --- | --- | --- | --- | --- |
| Siberian Husky | 24 | 2 | 10 | 12 | 0.7083 |
| Chinook | 3 | 2 | 1 | 0 | 0.1667 |

**b.**

| Breed | N | No haplotype | Heterozygote | Homozygote | Haplotype freq. |
| --- | --- | --- | --- | --- | --- |
| Siberian Husky | 66 | 7 | 18 | 41 | 0.7576 |
| Klee Kai | 2 | 0 | 2 | 0 | 0.5000 |
| Australian Shepherd | 41 | 36 | 5 | 0 | 0.0610 |
| Australian Cattle Dog | 16 | 15 | 1 | 0 | 0.0322 |
