# Supplementary material for: Direct-to-consumer DNA testing of 6,000 dogs reveals 98.6-kb duplication associated with blue eyes and heterochromia in Siberian Huskies
Source: PLoS Genet. 2018 Oct 4;14(10):e1007648. doi: 10.1371/journal.pgen.1007648 (PMC6171790; doi:10.1371/journal.pgen.1007648)
Supplement: S6 Table — (DOCX) [file pgen.1007648.s017.docx]

| ***a.*** | A^w^ | A^y^ | A^t^ | a |
| --- | --- | --- | --- | --- |
| A^w^ | 44 / 17 | 10 / 20 | 20 / 17 | 8 / 4 |
| A^y^ |  | 2 / 4 | 4 / 11 | 2 / 2 |
| A^t^ |  |  | 5 / 17 | 3 / 2 |
| a |  |  |  | 2 / 4 |

| ***b.*** | E | E^m^ | e |
| --- | --- | --- | --- |
| E | 35 / 34 | 8 / 23 | 35 / 21 |
| E^m^ |  | 1 / 4 | 4 / 11 |
| e |  |  | 18 / 6 |

| ***c.*** | B | b |
| --- | --- | --- |
| B | 37 / 57 | 51 / 38 |
| b |  | 12 / 4 |

| ***d.*** | K^B^ | K^y^ |
| --- | --- | --- |
| K^B^ | 0 / 0 | 12 / 34 |
| K^y^ |  | 88 / 66 |
