# Supplementary material for: Direct-to-consumer DNA testing of 6,000 dogs reveals 98.6-kb duplication associated with blue eyes and heterochromia in Siberian Huskies
Source: PLoS Genet. 2018 Oct 4;14(10):e1007648. doi: 10.1371/journal.pgen.1007648 (PMC6171790; doi:10.1371/journal.pgen.1007648)
Supplement: S1 Text — (DOCX) [file pgen.1007648.s018.docx]

**Supplemental Information**

*Other blue-eyed phenotypes.* Of the dogs in our original discovery panel with blue eyes not explained by either merle (*N* = 92 / 156) or the CFA18 marker (*N* = 41 / 156), before this panel was filtered to only those individuals with *log R* data for subsequent analyses, 35% were blue-eyed due to white facial markings (e.g. piebald) according to profile photos uploaded by their owners (N = 8). The remainder were merle cases not predicted by the CFA10 merle-associated SNP (*N* = 4), other eye colors misreported as blue (*N* = 3), or were unknown (*N* = 8 without profile photos). Of the five outlier dogs that did not appear to carry the duplication, despite being blue-eyed and carrying the CFA18 marker, four had atypical coat pigmentation (leucistic, mostly white, or unusual piebald-like cases). The fifth is a mixed breed dog with unexplained sectoral heterochromia, who exhibits elevated *log R* values for the last two markers within the duplicated sequence, possibly indicating a partial duplication.
